# Sensors and Actuators on Determining Parameters for Being Considered in Selection of Elastomers for Biomimetic Hands

**DOI:** 10.3390/s17061190

**Published:** 2017-05-23

**Authors:** Vlad Cârlescu, Dumitru N. Olaru, Gheorghe Prisăcaru, Cezar Oprişan, José Machado

**Affiliations:** 1 Department Mechanical Engineering, Mechatronics and Robotics, the “Gheorghe Asachi” Technical University of Iasi, Bulevardul Profesor Dimitrie Mangeron 67, 700050 Iași, Romania; vlad.carlescu@tuiasi.ro (V.C.); dolaru@mail.tuiasi.ro (D.N.O.); prisacaru_ghe2004@yahoo.com (G.P.); coprisan@mail.tuiasi.ro (C.O.); 2 Mechanical Engineering Department, MEtRICs Research Center, University of Minho, Campus of Azurém, 4800-058 Guimarães, Portugal

**Keywords:** human finger, Young’s modulus, indentation speed, viscoelastic behavior, biomimetic hand, dissipated power

## Abstract

In this paper, the authors present a new methodology to study the viscoelastic behaviour of the human finger. The methodology is based on experimental research consisting of a finger’s indentation with a small steel cylindrical object for various indentation speeds. The tests were realized on a CETR UMT-2 Tribometer for indentation speed between 0.02 mm/s and 4 mm/s with a normal load of up to 22 N. Using the force–deformation diagrams recorded at the smallest indentation speed determined the elastic modulus of the human finger according to an adapted Hertzian model. By considering the increasing of the indentation force with indentation speed, the viscous component of the human finger was evidenced. The power dissipated in the finger tissue as a result of the prehension process has been obtained as a function of indentation speed and indentation depth. In addition, a general equation for the prehension force as a function of indentation speed and indentation depth has been obtained. The results of this study will be relevant for selection of the specific elastomers used in biomimetic hands.

## 1. Introduction

The prehension of the small objects realized by a biomimetic hand is dependent on the mechanical behavior of the elastomers that cover the solid structures of the artificial fingers [1]. Both the friction/adherence and elastic deformations of the elastomer layers are important mechanical properties to assure a good prehension of the small objects by the biomimetic hands [1]. The natural human fingers are adapted for prehension for a lot of the small and large objects and are the best source for inspiration in realization of the biomimetic hands. The human fingers are covered with a complex skin composed of three layers with different mechanical properties: epidermis, dermis and subcutaneous fat [2]. In the prehension process, the skin is considered as a viscoelastic material with different mechanical properties in lateral and vertical directions that depend on the loading conditions and indentation depth [2].

A lot of publications studied experimentally the human skin friction and propose various models to evaluate the friction coefficients between human skin and various surface materials [3,4,5,6,7]. Derler et al. [3] investigated the friction behavior of the index human skin in contact with smooth and rough glass surface under dry and wet conditions. Derler et al. [3] obtained values for friction coefficients between 0.15 and 1, as a function of contact pressure according to the follow function, where *p* is contact pressure and the exponent α have values between 0.15 and 0.96 in the dry finger/glass contacts. Derler and Rotaru [4] established by experiments for the index finger sliding on a wet, smooth glass pad that a friction coefficient can be expressed as a function of normal force *Fn* according to the following relationship. Tomlinson [5,6] investigated the friction between human fingers and flat, rough or textured surfaces for a lot of materials (glass, steel, plastics, rubber). Tomlinson [5] demonstrated that the total friction force between the finger and a flat surface is a sum of adhesive forces, skin deformation forces, and capillary forces.

Interesting experimental investigations, regarding the friction coefficient between the human finger and a rough surface, have been recently realized by Zhang et al. [7].

One of the most important mechanical parameters is the Young modulus of the skin. Therefore, Kwiatkowska et al. [2] determined the Young modulus of the skin by using the Hertz model between the elastic deformation of the forearm human skin indented with steel balls of 2 mm and 5 mm. For the normal load between 0.19 N and 0.5 N, the authors obtained values for skin effective Young modulus between 0.03 and 0.12 MPa, with decreasing of the Young modulus with increasing of the ball diameter, and increases with the normal load for a constant probe radius. In his PhD thesis, Liu [8], by using the penetration of the finger skin with a small ball obtained the stiffness of the skin as the ratio between variation of applied load *dFn* and variation of elastic deformation *dδ*. According to the Oliver and Pharr [9] model, Liu determined the global Young’s modulus of the skin with equation: $E_{skin}=S_{skin}\cdot \sqrt{\pi }/2\cdot \sqrt{A}$, where *A* is the projected contact area between the ball and skin. Han et al. [10] evaluated the contact area between a human skin finger and a plane surface and proposed the following equation for the contact area *A*: $A=k\cdot F_{n}^{b}$, where *k* and *b* are constants determined by the experiments.

Kuilenburg et al. [11] studied the effective elastic modulus of the skin from micro to macro scale by using the indenter method of the skin. Therefore, the authors obtained values from 0.15 MPa to 0.015 MPa when the radius of curvature of the indenter increases from 10  µm to 10  mm.

By indenter of the human skin with a steel ball having a 15 mm radius, Morales Hurtado [12] obtained the variation of the effective elastic modulus of the skin between 0.035 MPa and 0.06 MPa.

Zahouani et al. [13] studied the human skin rheology using a dynamic indentation device. Based on the Kelvin–Voigt viscoelastic model, the authors solved the differential equation for small vibration of the human skin induced by a sine displacement applied to the skin surface with a spherical indenter. The authors measured simultaneously the force and displacement and obtained the stiffness and the damping properties of the indenter/skin contact. A complex modulus, including a real component corresponding to Young’s storage modulus and an imaginary component corresponding to the Young’s loss modulus, has been obtained by the authors. For a dynamic indentation at 10 Hz frequency and with a vibrating mass having 4.2 g, the determined complex modulus varied between 0.007 MPa and 0.01 MPa depending on the age.

By analyzing a lot of theoretical and experimental results presented in literature, Derler and Gerhardt [14] concluded that elastic moduli of human skin in vivo varying over 4–5 orders of magnitude, between 0.004 MPa and 57 MPa, depending on the measurement procedures, anatomical site, skin hydration level, human age, and individual person. To evaluate the friction coefficient between a steel ball and forearm skin, Derler and Gerhardt [14] determined a mean pressure in ball–skin contact by using the Hertz model and considering the elastic modulus for the value of 0.040 MPa and a Poisson ratio of 0.49.

Barnea et al. [15,16] developed a new methodology to evaluate the mechanical properties of the human finger’s skin by using the indentation with a small rigid cylinder. The contact deformation between cylinder and finger has been solved by using the Hertz model and the Young’s modulus of the finger, being adopted from literature. Because the Young modulus of the human skin is a complex parameter and has a large variation in literature depending on the determined method, normal load, age, and humidity, Oprisan et al. [17] developed a new methodology to determine the effective Young’s modulus of the finger skin. The authors’ methodology is based on the equivalence between measured indented deformation of the finger’s skin and calculated of the indented deformation by using Hertzian contact model. The results obtained by Oprisan et al. [17] indicate an increasing of the effective Young’s modulus by an increasing of the indenter’s force. Excepting the indentation with a sine displacement function [13], the other indentation methods adopted a constant or variable normal force with imposed indentation speed, with a lot of indentation conditions.

If the sensors control the normal and tangential prehension forces, the necessary power for the prehension, in correlation with the prehension forces and prehension speed, is an important parameter for adaptation of the adequate micro motors. To make evident both the viscoelastic behavior of the finger skin and the power dissipated in the finger indentation, the authors propose, in the present paper, a new methodology based on the evaluation of the indentation force as a function of indentation speed. Thus, by using indentation speed between 0.02 mm/s and 4 mm/s has made evident the increasing of the damping effect of the skin by an increasing of the indentation speed and, as a consequence, an important increasing of the indentation power loss.

An original dependence of the power loss as a function of the indentation speed and indentation depth for a human finger has been obtained.

In addition, an original dependence on the indentation force as a function of the indentation speed and indentation depth for a human finger has been obtained.

## 2. Experimental Investigations

**Figure 1:**
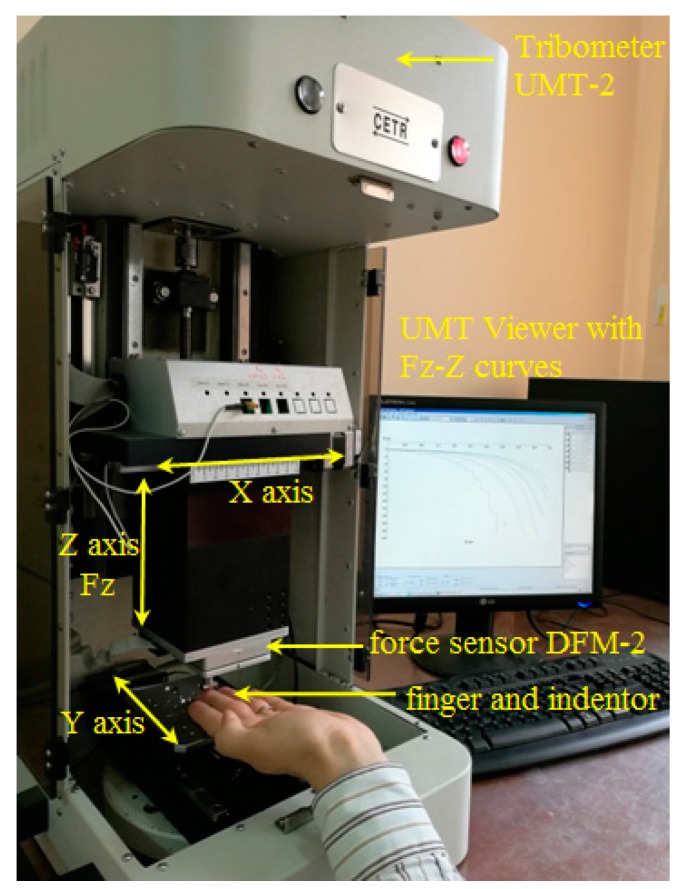
General view of the experimental equipment.

**Figure 2:**
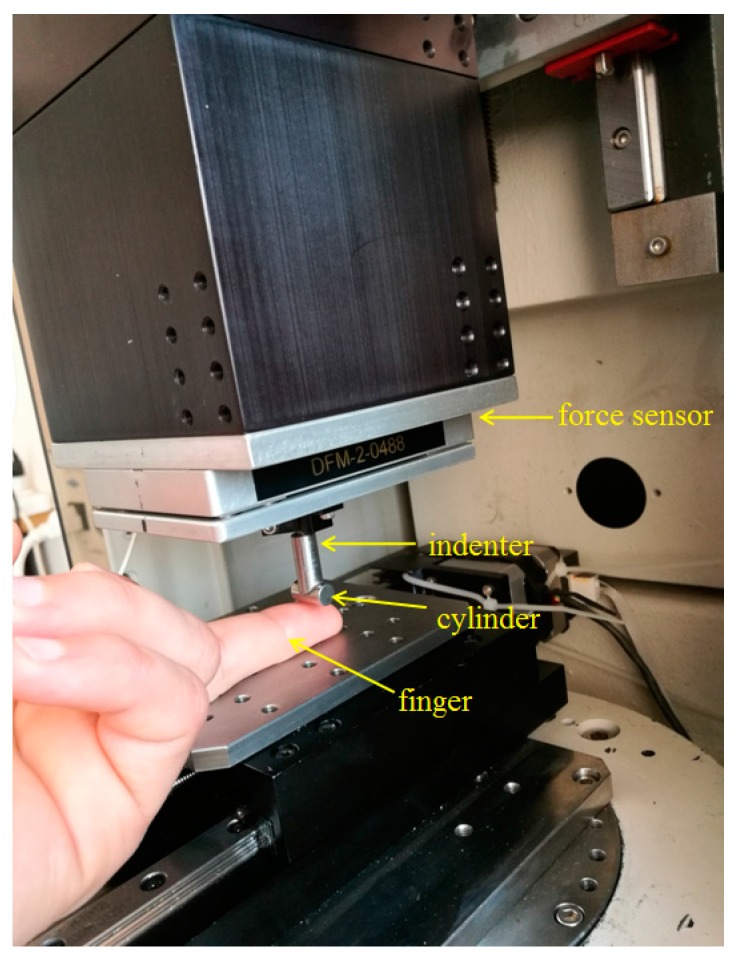
Detailed indentation of the middle finger with the steel cylinder.

The indentation experiments were realized by using the Tribometer CETR UMT-2 (Universal Micro-Materials Tester) from the Tribology laboratory of the Mechanical Engineering Faculty from Iasi, Romania. Figure 1 presents a general view of the testing equipment and procedure. The Tribometer is equipped with a controlled force sensor DFM-2 (Dual Friction/Load Sensor Medium Range) having the possibility to determine the normal force *Fz* acting on the middle finger by a steel cylinder fixed in the top of a pin. Figure 2 presents details of the contact between the steel cylinder and the middle finger. The cylinder has a diameter of 7 mm, a length of 14 mm and a surface roughness of *Ra* = 0.06 μm. On the *z*-axis (vertical direction) are imposed indentation speeds between 0.02 mm/s and 4 mm/s. In addition, a maximum indentation depth of the finger of 4 mm is fixed. The resulting forces obtained in indentation processes for every indentation speed are registered on the tribometer’s computer and are dependent on the indentation speed. The start of the force’s register is considered in the moment of the touch of the finger by the cylinder. The middle finger is supported by the table of the tribometer (see Figure 2). The time of the indentation is dependent on the indentation speed and varied between 1 and 200 s. The tests were performed for a dry middle finger for a young 30-year-old man.

## 3. Determination of the Effective Young’s Modulus of the Middle Finger

The viscoelastic behavior of the human skin has been made evident and evaluated by a lot of authors, and some are presented in the introduction. Based on the Kelvin–Voigt viscoelastic model used by Zahouani et al. [13], the total indentation force *Fz* has two components: a component generated by the elastic behavior, *Fz,e*, and a component generated by the damping caused of the internal losses in the human finger tissues, *Fz,v*:

(1) Fz=Fz,e+Fz,v

Zahouani et al. [13] proposed a dynamic methodology based on a sine indentation force imposed on a ball–finger skin by solving a differential equation and determined two components of the complex Young’s modulus.

We propose a new methodology to determine the two components of the Equation (1) and the effective elastic Young’s modulus of the human finger.

Therefore, based on the Ex-Poro-Hydrodynamic theory developed by Pascovici et al. [18] in porous materials soaked with liquid under the compression, the damping force increases with indentation speed. By similarity, the finger tissue includes a lot of blood capillaries and by increasing of the indentation speed, it is normal that the damping force increases. The preliminary indentation’s experiments of the finger with various speeds confirmed our initial idea. Thus, in Equation (1), the damping force *Fz,v* must be a variable force as a function of the indentation speed.

For a very low indentation speed, we can consider that the damping component can be neglected and the total measured indenter force *Fz* is caused only by the elasticity of the finger tissue, *Fz,e*.

**Figure 3:**
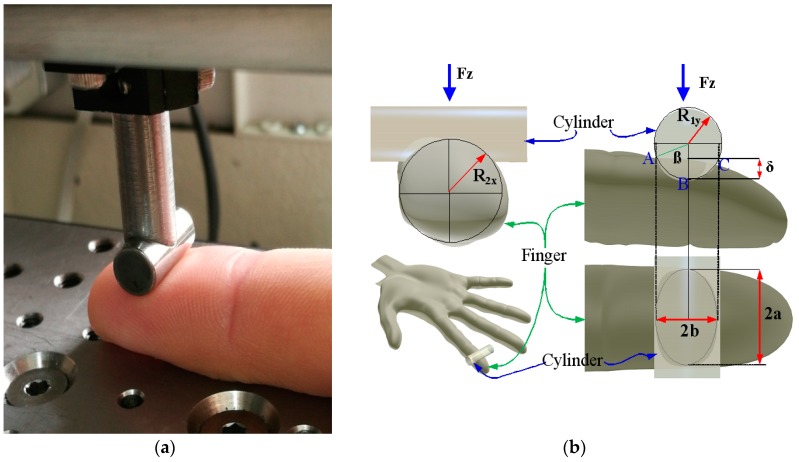
In vivo contact cylinder—middle finger (**a**); the geometrical parameters and Hertz deformation in the contact between cylinder and middle finger (**b**) [17].

To determine the Young’s modulus of the finger’s skin, the methodology presented by Oprisan et al. in [16] was adapted. Thus, for the contact deformation between the cylinder and the middle finger *δ*, the Hertz equation has been used:

(2) $$\delta =\delta^{*}{\left\{\frac{3\cdot Fz}{2\sum \rho }\cdot \left[\frac{1-\nu_{c}^{2}}{E_{c}}+\frac{1-\nu_{f}^{2}}{E_{f}}\right]\right\}}^{\frac{2}{3}}\cdot \frac{\sum \rho }{2},$$

where *E_c_*, *E_f_* are the Young’s modulus for the cylinder and for the finger, respectively, and *ν_c_*, *ν_f_* are the Poisson coefficients for the cylinder and for the finger, respectively. Curvature sum *Σρ* and geometrical dimensionless parameter δ* have been determined by Oprisan et al. in [16], according to the geometry of the finger and the diameter of the cylinder. The following values for the finger’s radii presented in Figure 3b have been used: transversal radius *R*_2*,x*_ = 8 mm and a longitudinal radius *R*_2*,y*_ = 30 mm. Because the Young’s modulus of the steel is about 2.1 × 10^5^ MPa and the Young’s modulus of the human skin is less than 1 MPa, and imposing the Poisson coefficient of the finger value of 0.5, Equation (2) becomes:

(3) $$\delta =0.308\times {\left(Fz/E_{f}\right)}^{2/3},$$

where *Fz* is included in N, *E_f_* is included in MPa and *δ* results in mm. To determine the Young’s modulus, Oprisan et al. [16] elaborated a simple methodology to realize the fitting of the experimental curve of the elastic deformation of the skin’s finger *δ* with Equation (3) in the vicinity of the various indentation force *Fz*. In [16], the authors applied the above-mentioned methodology for various values of the normal forces *Fz*, and obtained an increasing of the Young‘s modulus between 0.04 MPa and 0.24 MPa, for increasing of the normal forces between 0.2 N and 10 N, respectively.

In the present paper, we determined the variation of the Young’s modulus as a function of the indentation force by imposing a minimum indentation speed of 0.02 mm/s and a maximum indentation deformation *δ* of 4 mm.

## 4. Experimental Results

### 4.1. Determination of the Young’s Modulus

**Figure 4:**
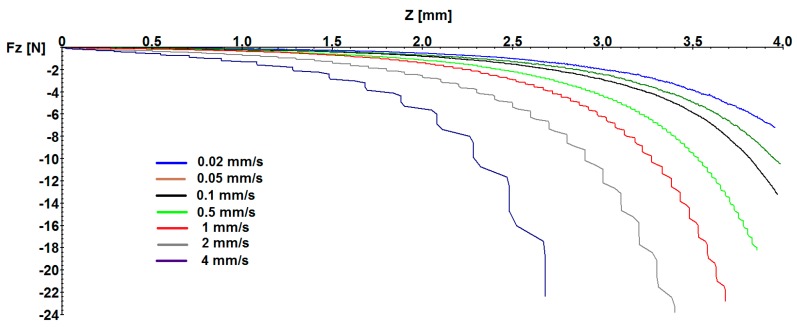
Variation of the total indenter force *Fz* as a function of indentation speed from 0.02 mm/s to 4 mm/s.

The variation of the indentation force *Fz* measured by the sensor in the indentation processes on the Tribometer UMT-2, for various indentation speeds, is presented in Figure 4.

In Figure 4, the values of the indentation force *Fz* are negative, according to the coordinate system of the Tribometer and *Z* represents the contact deformation *δ* of the finger.

**Figure 5:**
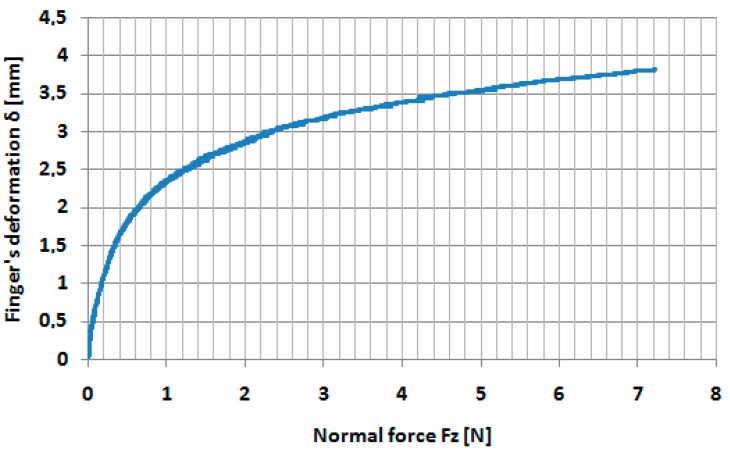
Variation of the finger’s deformation with normal force *Fz* for indentation speed *v* = 0.02 mm/s.

As we supposed, by increasing of the indentation speed, the normal force *Fz* increases as a result of the damping effect in the finger’s tissue. It can be observed that the minimum indentation forces *Fz* are obtained for the minimum indentation speed *v* = 0.02 mm/s. The variation of the deformation *δ* as a function of the indentation force *Fz* has been presented in Figure 5, for the minimum indentation speed *v* = 0.02 mm/s.

**Figure 6:**
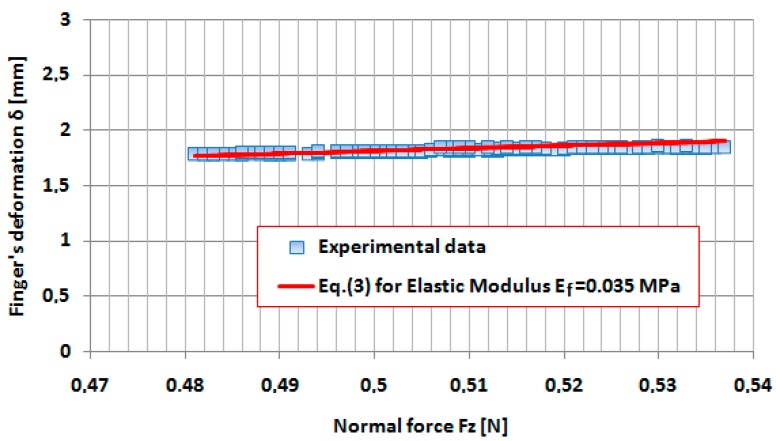
Variation of the finger’s deformation *δ* in the vicinity of the applied force *Fz* = 0.5 N both for the experiment and for Equation (3) with *E_f_* = 0.035 MPa.

**Figure 7:**
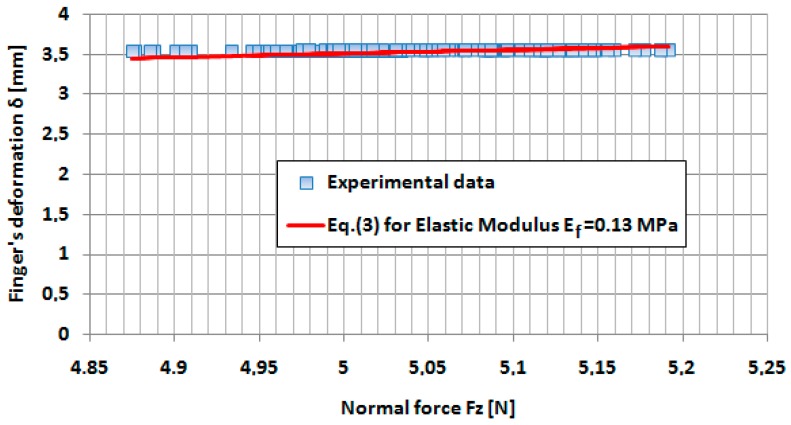
Variation of the finger’s deformation *δ* in the vicinity of the applied force *Fz* = 5 N both for the experiment and for Equation (3) with *E_f_* = 0.13 MPa.

We supposed that, at this very speed indentation (*v* = 0.02 mm/s), the damping effect in the finger tissue can be neglected and the finger deformation is dominant elastic. According to the methodology presented in [17], the variation of the Young’s modulus was determined with the indentation force *Fz*, by fitting the deformation–force curves in the vicinity of the indentation force from 0.1 to 7 N. In Figure 6 and Figure 7, the fitted curves in the vicinity of the forces 0.5 and 5 N, respectively, are presented.

**Figure 8:**
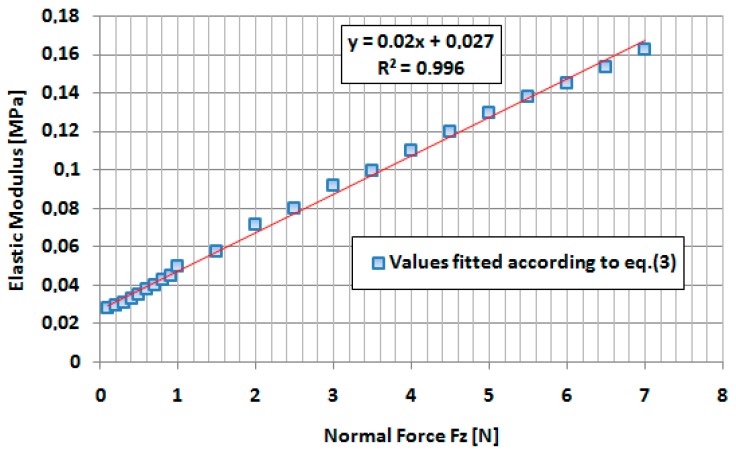
Variation of the Young’s modulus of the middle finger as a function of the indentation force *Fz*.

The variations of the Young’s modulus, as a function of the indentation force according to the fitting procedure, are presented in Figure 8. A very good linear approximation has been obtained and the dependence between Young’s modulus of the finger and indentation force *Fz* applied by the cylinder can be estimated by the following equation [MPa]:

(4) $$E_{f}(Fz)=0.02\times Fz+0.027,$$

where *Fz* is expressed in N.

By including Equation (4) in Equation (3), the following equation for variation of the elastic deformation of the finger with indentation force *Fz* was obtained:

(5) $$\delta =0.308\times {\left[\frac{Fz}{0.02\times Fz+0.027}\right]}^{2/3}.$$

**Figure 9:**
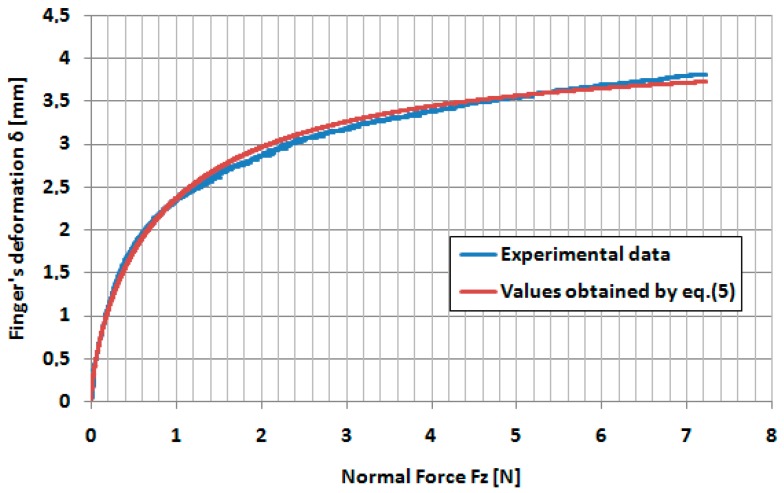
Correlation between experimental data and theoretical model for the dependence between finger’s deformation and normal force *Fz*.

The correlations between the experimental values of the finger’s deformation as a function of indentation forces and the theoretical model given by Equation (5) are presented in Figure 9.

It can be seen that a very good correlation between experimental values of the finger’s deformation and the finger deformation determined by the Equation (5) has been obtained.

From Equation (5), the variation of the indentation force *Fz* as a function of indentation deformation *δ* can be obtained. This force can be considered as an elastic component in Equation (1) *Fz,e*, and has the following equation:

(6) $$Fz,e=0.027\times \left[\frac{\delta^{1.5}}{0.171-0.02\times \delta^{1.5}}\right].$$

A nonlinear dependence between the applied force and indentation deformation *δ* can be obtained.

### 4.2. Evaluation of the Power Loss in the Indentation Process

**Figure 10:**
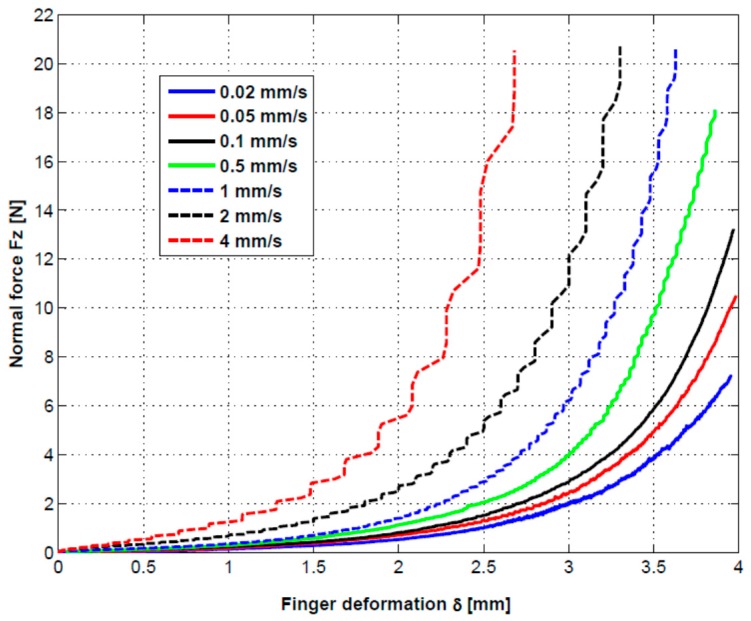
Variation of the experimental indentation force *Fz* as a function of indentation speed.

The experimental dependences between indentation force *Fz* and indentation deformation *δ* proved a strong influence of the indentation speed as it can be observed in Figure 10, obtained from the numerical values given in the indentation process with the Tribometer. For the indentation forces *Fz*, positive values have been imposed.

The following important observations can be made in relation with the experimental results presented in Figure 10:

1. By increasing of the indentation depth, the indentation force *Fz* has a continual increase. It is a normal process as a result of the compression of the finger’s tissue.
2. By increasing of the indentation speed, for some indentation depth, the indentation forces have important increases as result of a complex damping effect caused by the resistance of the finger’s tissue to have a fast response corresponding to the speed of the indentation.
3. The damping process is a complex one and depends on both the indentation depth and the indentation speed. It is clear that the power loss dissipated in the finger tissue increases with increasing of the indentation speed.

The total power dissipated in the indentation process for every indentation speed has been determined according to the following equation:

(7) $$P=\frac{1}{t}\times \sum_{1}^{n}Fz,i\times \left(\delta_{i+1}-\delta_{i}\right),$$

where *Fz,i* are the values of the indentation force at the step *i* in N and (*δ*_*i+*1_ *− δ_i_*) are the differences of the indentation depth for every step in mm. The number of the steps *n* varies with every indentation speed, from approx. 19,000 steps at the 0.02 mm/s to 150 steps at 4 mm/s. 

In Equation (7), *t* is the time during the indentation process, calculated for every indentation speed with equation:

(8) $$t=\frac{\delta_{\max}}{v},$$

where *δ*_max_ is the maximum indentation depth in mm, obtained for every indentation speed, and *v* is the indentation speed in mm/s. As it can be seen in Figure 10, the maximum indentation depth *δ*_max_ varied between 4 mm and 2.7 mm as a function of indentation speed.

**Figure 11:**
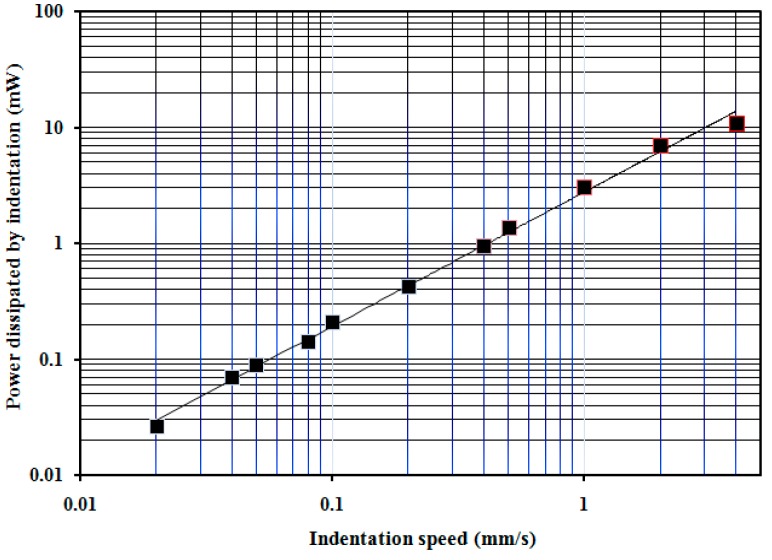
Variation of the total power dissipated in the indentation of the finger as a function of indentation speed.

The total power dissipated in the indentation process has been determined according to Equations (7) and (8) and are presented in Figure 11 as black points as a function of indentation speed. By curve fitting the experimental values, the following dependence has been obtained [mW]:

(9) $$P(v)=2.769\times v^{1.157}.$$

As is obtained and presented in Figure 11, the total dissipative power in the finger’s tissue has a value of 0.032 mW for the minimum indentation speed of 0.02 mm/s, while the total dissipative power in the finger’s tissue has a value of about 12 mW for the indentation speed of 4 mm/s, which means more than two orders of magnitude. These results confirm our supposition that a very low indentation speed for the dissipative processes can be neglected. In the indentation process, the variation of the cylinder speed from zero to imposed values was dependent on the damping effect in the finger tissue. Thus, for the indentation speed of 0.02 mm/s, the time for increasing of the speed from zero to the value of 0.02 mm/s was 1.6 s, which means an acceleration of 0.013 mm/s^2^. For the indentation speed of 4 mm/s, the time for increasing of the speed from zero to the value of 4 mm/s was 0.5 s, which means an acceleration of 8 mm/s^2^. In addition, the time to the indenter for the finger tissue was 200 s for the speed of 0.02 mm/s, while the time to the indenter for the finger tissue was 0.97 s for the speed of 4 mm/s. If we compare an indentation process with an acceleration of 0.013 mm/s^2^ during 200 s with one having an acceleration of 8 mm/s^2^ during 0.97 s, it can be accepted that the damping effect can be neglected at the indentation speed of 0.02 mm/s. 

In Figure 11, only the variation of the total power dissipated in the finger’s tissue for some imposed speeds (from 0.02 mm/s to 4 mm/s) are presented, considering that each test’s indentation time was calculated from Equation (8).

Equation (9) can be used only for the total power dissipated during the time determined with Equation (8). It is clear that if we impose another indentation depth, Equation (7) has other limits and the power loss will have another value than indicated in Figure 11.

Thus, the power dissipated in the indentation process depends on both the speed and on the indentation depth.

**Figure 12:**
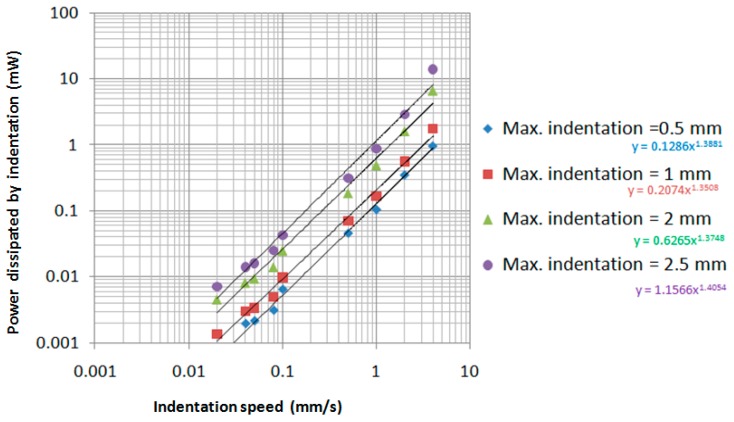
Variation of the total power dissipated in the indentation of the finger as a function of indentation speed and maximum indentation.

In Figure 12, the variation of the power dissipated by indentation, as a function of indentation speed, and indentation depth are presented. The same Equation (7) has been used but the limits for summing were reduced according to the imposed maximum indentation *δ*_max_ (0.5 mm, 1 mm, 2 mm and 2.5 mm).

It can be seen that, by increasing of the indentation depth, the power dissipated increases.

By curve fitting of the power values for the some indentation value, we obtained a power low having the following general equation:

(10) $$P(\delta ,v)=P_{1}(\delta )\times v^{\left(1.37...1.4\right)}.$$

The function *P*_1_(*δ*) was obtained by curve fitting of the numerical values obtained in the power lows for every indentation depth, and the following exponential function was the result:

(11) $$P_{1}(\delta )=0.0747\times e^{1.08\cdot \delta }.$$

From Equations (10) and (11), the following general equation was the result for the dissipated power [mW] in the finger’s tissue as a function of depth of indentation *δ* and indentation speed *v*:

(12) $$P(\delta ,v)=\left(0.0747\times e^{1.08\cdot \delta }\right)\times v^{1.385},$$

where depth of indentation *δ* is given in mm and indentation speed *v* is given in mm/s.

From Equation (6), the power dissipated in the finger’s tissue caused by the “elastic hysteresis” *Pe(δ)* [mW] can be obtained:

(13) $$P_{e}(\delta )=\frac{1}{t}\times \int_{0}^{\delta }0.027\times \left[\frac{\delta^{1.5}}{0.171-0.02\times \delta^{1.5}}\right]\times d\delta .$$

Equation (12) is a general one including both elastic deformation and damping effect caused by the speed and can be used in the limits imposed in the experiments, while Equation (13) is a particular case when the damping effect can be neglected at a very low indentation speed.

The presence of the indentation depth *δ* and indentation speed *v* in the general Equation (12) for variation of the power loss suggest to us that a simple Kelvin–Voigt viscoelastic model is difficult to adopt for our indentation procedure.

A complex equation for the indentation force as a function both the indentation depth *δ* and indentation speed *v* was obtained by curve fitting the force-deformation curves from Figure 10.

Thus, every force-deformation curve can be approximated by exponential functions having the following general form:

(14) $$F\left(v,\delta \right)=F_{1}\left(v\right)\times e^{F_{2}\left(v\right)\cdot \delta },$$

where *F*_1_(*v*) and *F*_2_(*v*) are obtained by curve fitting the numerical values for indentation speeds between 0.02 mm/s and 4 mm/s.

**Figure 13:**
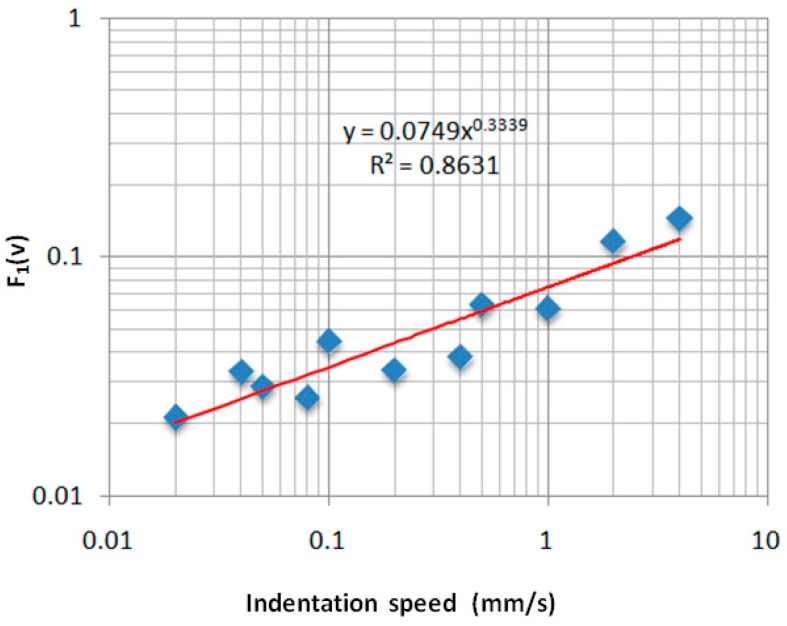
Variation of the *F*_1_(*v*) as a function of indentation speed.

**Figure 14:**
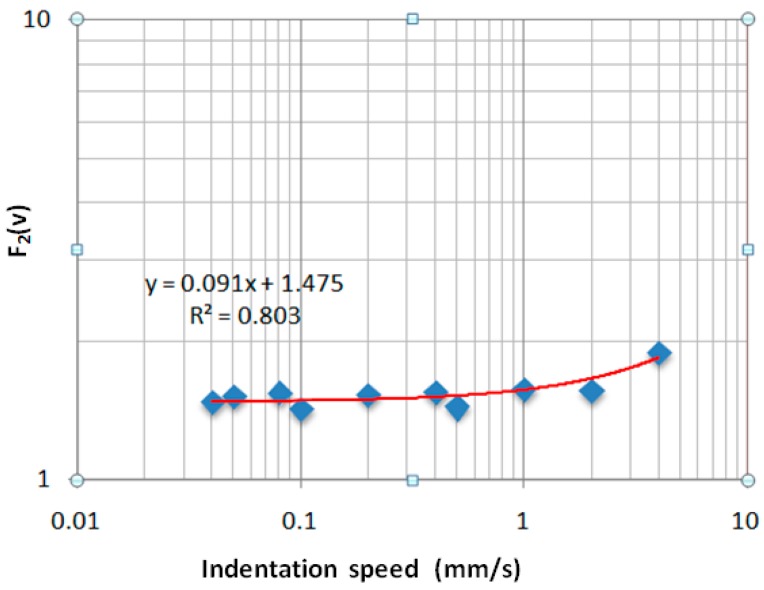
Variation of the *F*_2_(*v*) as a function of indentation speed.

In Figure 13 and Figure 14, the curve fitting of the data are presented for *F*_1_(*v*) and *F*_2_(*v*), respectively. The blue points represent the numerical values for various indentation speeds and the red curves are the analytical model.

The following equations for *F*_1_(*v*) and *F*_2_(*v*) have been obtained:

(15) $$F_{1}\left(v\right)=0.0749\times v^{0.3339},$$

(16) $$F_{2}\left(v\right)=0.0919\times v+1.4757.$$

It must be highlighted that if Equation (14) has a general form, Equations (15) and (16) include numerical values obtained as a result of the experiments on a middle finger of young people where indentation speed is given in mm/s and the indentation force *F*(*v,δ*) is obtained in N.

The general equation for the indentation force between a small steel cylinder and a human finger given by Equation (14) suggests the complexity of the dependence between the prehension force and the two parameters: depth of indentation and indentation speed.

The two Equations (12) and (14) can be considered as starting points for new experiments by considering the prehension of the other small objects by the human fingers. Based on these results, the authors intend to extend the similar research applied to some classes of the elastomers used in the construction of the biomimetic hands.

## 5. Conclusions

The authors determined the Young’s modulus for a middle human finger by using the indentation of the finger with a small steel cylinder at a very low indentation speed to avoid the dissipative processes in the finger’s tissue. The Hertz contact model has been used and it has been obtained a linear dependence between the Young’s modulus of the human finger and indentation force, for a maximum indentation depth of 4 mm. Values for Young’s modulus between 0.027 MPa and 0.16 MPa have been obtained. In addition, a general equation between indentation force and elastic deformation of the finger has been obtained (Equation (6)).

To determine the influence of the indentation speed on the viscoelastic behavior of the finger’s tissue, the authors performed indentations between steel cylinder and human middle finger for a lot of indentation speeds between 0.02 mm/s and 4 mm/s. By increasing of the indentation speed, an important increase of the indentation forces has been obtained. Thus, the increase of the damping effect in the human finger’s tissue was made evident by increasing of the indentation speed.

To make the dissipative effect in the finger’s tissue evident as a result of increasing of the indentation speed, the authors determined the total power dissipated during the indentation process for every indentation speed. When the indentation speed increased between 0.02 mm/s and 4 mm/s, the total power dissipated in the finger’s tissue increased from 0.032 mW to 12 mW, respectively.

Based on the experimental values and by curve fitting the experimental results, the authors obtained a general equation for power dissipated in the finger’s tissue as a function of indentation depth and indentation speed (Equation (12)). The power dissipated in the finger’s tissue by using the contact between steel cylinder and human finger can be expressed as a product between a function depending on the indentation depth and a function depending only on the indentation speed.

It was demonstrated that it is difficult to adopt a simple Kelvin–Voigt viscoelastic model for the indentation procedure. A complex equation for the indentation force as a function of both indentation depth *δ* and indentation speed *v* was obtained by curve fitting the force–deformation curves obtained by experiments.

The authors consider that the results highlight the importance of the indentation speed in the description of the viscoelastic behavior of the finger’s tissue. These kinds of tests and consequent results are not found in the literature.
